# Incident reviews in UK maternity units: a systematic appraisal of the quality of local guidelines

**DOI:** 10.1186/s12884-015-0483-6

**Published:** 2015-03-14

**Authors:** Anjali Shah, Olaa Mohamed-Ahmed, Philippe Peirsegaele, Charlotte McClymont, Marian Knight

**Affiliations:** National Perinatal Epidemiology Unit, University of Oxford, Old Road Campus, OX3 7LF Oxford, UK

**Keywords:** Maternity, Incident, Review, AGREE, Guidelines, UK, National, Survey

## Abstract

**Background:**

Maternity care is recognised as a particularly high-risk speciality that is subject to investigation and inquiry, and improvements in risk management have been recommended. However, the quality of guidelines for local reviews of maternity incidents is unknown. The aim of the study is to appraise the quality of local guidance on conducting reviews of severe maternity incidents in the National Health Service.

**Methods:**

Guidelines for incident reviews were requested from all 211 consultant-led maternity units in the UK during 2012. The Appraisal of Guidelines for Research and Evaluation Instrument (AGREE II) was used to evaluate the quality of guidelines. The methods used for reviewing an incident, the people involved in the review and the methods for disseminating the outcomes of the reviews were also examined.

**Results:**

Guidelines covering 148 (70%) of all NHS maternity units in the UK were received for evaluation. Most guidelines (55%) received were of good or high quality. The median score on ‘scope and purpose’ (86%), concerned with the aims and target population of the guideline, was higher than for other domains. Median scores were: ‘stakeholder involvement’ (representation of users’ views) 56%, ‘rigour of development’ (process used to develop guideline) 34%, ‘clarity of presentation’ 78%, ‘applicability’ (organisational and cost implications of applying guideline) 56% and ‘editorial independence’ 0%. Most guidelines (81%) recommended a range of health professionals review serious maternity incidents using root cause analysis. Findings were most often disseminated at meetings, in reports and in newsletters. Many guidelines (69%) stated lessons learnt from incidents would be audited.

**Conclusions:**

Overall, local guidance for the review of maternity incidents was mostly of good or high quality. Stakeholder participation in guideline development could be widened, and editorial independence more clearly stated. It was unclear in over a quarter of guidelines whether changes in practice in response to review recommendations were audited or monitored; such auditing should be mandatory. Further research is required to examine the translation of guidance into practice by evaluating the quality of local reviews of maternity incidents.

## Background

Learning from clinical incidents is a recognised part of ongoing quality improvement in health care [1-3]. Many factors affect quality of care including the organisation of services, leadership, monitoring systems, adequate infrastructure, the resources available, both human and material, and continual improvement. Maternity care is recognised as a particularly high-risk speciality that is subject to investigation and inquiry, and improvements in risk management have been recommended [4-6]. Quality improvement requires timely and high quality data on health outcomes, as identified and recommended by the World Health Organisation [3].

Maternal deaths are rare in the UK, and thus reviews of other severe complications of pregnancy and the puerperium can provide an additional perspective to help learn lessons to improve care [5]. The National Reporting and Learning System [7], currently administered by the Care Quality Commission and formerly by the National Patient Safety Agency, plays an integral role in monitoring commonly-occurring errors and disseminating feedback nationwide. However, it is recognised that the number of cases of specific incidents reported to this service are considerably fewer than the number of events occurring in practice [8,9]. This may represent a mismatch between the incidents viewed locally as important to review, and those recommended at a national level. Maternal deaths are not always cited on lists of incidents triggering local reviews in UK maternity units, and sepsis only appears on 64% of these lists despite prevention of this condition being an international priority [10-12]. The definition of a maternity ‘incident’ that should trigger a review thus varies across the UK [12].

Guidance exists from both professional organisations, and national bodies [1,13-16] on tools for reviewing incidents at the local level, such as root cause analysis [17]. A study of the transfer of women from midwifery units to obstetric units during labour found considerable variation in local guidelines, and many were judged to be of poor quality [18].

The aims of this study were to systematically appraise the quality of guidance on conducting local incident reviews in maternity units in the UK and to describe how incidents are reviewed, how findings are disseminated and the processes in place to ensure lessons are learned for future care.

## Methods

All 211 consultant-led maternity units in the UK were contacted up to three times, by postal mail, e-mail and telephone, and asked to supply a copy of their maternity risk management strategy and/or their incident review procedure during 2012.

For every maternity unit in England we noted the level achieved in the Clinical Negligence Scheme for Trusts (CNST) [13], which is only applicable in England, and compared the levels for those units that participated in the study and those that did not. A CNST level of 1 indicates that a unit has a process for managing risks that has been documented, a CNST level of 2 indicates that a unit has been assessed as following the process, and a CNST level of 3 indicates that a unit monitors the process for managing risk, identifies deficiencies and draws up action plans to reduce risks.

### Quality appraisal

The Appraisal of Guidelines for Research and Evaluation (AGREE) II instrument was used for assessing the quality of clinical practice guidelines [19]. Each guideline was assessed using 23 items organised into six domains. Each domain relates to a different dimension of guideline quality.

1. Scope and purpose (three items). This focuses on the overall objective of the guideline, the specific health questions and the target population. For this study ‘types of risk or incident’ were considered in place of specific health questions.
2. Stakeholder involvement (three items). This is concerned with whether the guideline was developed by the appropriate stakeholders.
3. Rigour of development (eight items). This relates to the process used to gather and synthesise the evidence, the methods to formulate the recommendations, and the procedure for updating them.
4. Clarity of presentation (three items). This focuses on the structure, format and ease of understanding the guideline.
5. Applicability (four items). This relates to likely facilitators and barriers to implementation, advice and tools for use, resource implications of applying the guidelines and monitoring and/or auditing criteria.
6. Editorial independence (two items). This is concerned with the independence of the guidelines from the funding body and whether any competing interests of guideline development group members have been recorded and addressed.

Each item was scored on a seven-point Likert scale ranging from 7 ‘Strongly agree’ to 1 ‘Strongly disagree’ with a mid-point of 4. An overall assessment of the quality of the guideline was made by calculating the mean average score from the 23 scores for each AGREE criteria. Any score of 0-2.9 was classified as ‘poor quality’ , a score of 3-3.9 was ‘average quality’ , a score of 4-4.9 was ‘good quality’ and a score of five or more was considered to be ‘high quality’.

Prior to evaluating any guidelines, the two researchers (an epidemiologist and a public health doctor) met to discuss and agree how best to apply each question within the AGREE II criteria to incident reporting within maternity services. Outstanding queries were resolved by a midwife and clarifications for several questions were noted. The two researchers appraised each guideline independently using the AGREE II method. If the score for any item differed by more than two points between the appraisers, they met to discuss their scores. Scores were revised when errors or inconsistencies in interpretation of guidelines were identified. Analyses were based on the revised scores.

To assess the content of each guideline, four additional issues were considered when appraising each guideline: the method(s) used for reviewing an incident, the types of professionals involved in the review, the methods for disseminating the outcomes and whether units had a process to audit changes in clinical practice arising from incidents.

### Analysis

The presence of response bias was assessed by comparing the trusts that returned guidelines with those that did not using a t-test. The comparisons made were the number of births in each maternity unit in the UK in the year 2011, and the CNST level for units in England that rates compliance with Maternity Clinical Risk Management Standards [13].

Standardised domain quality scores for each guideline were calculated according to the AGREE II instrument standard methods. The two appraisers’ scores were summed and standardised by scaling the total as a percentage of the maximum possible score for that domain. The scaled domain score is calculated as ((obtained score-minimum possible score)/(maximum possible score-minimum possible score)) × 100. Thus the possible range for standardised domain scores is 0-100%. The AGREE II instructions state that domain scores are independent and should not be aggregated into a single quality score [19].

Median domain scores with 95% CI and the proportion of guidelines scoring less than 30%, 30-60%, and more than 60% were calculated. Scores for the lowest scoring and highest scoring domains were compared with scores for other domains using the Wilcoxon matched pairs signed rank sum test for non-parametric data.

### Ethics committee approval

This work is classified as audit and therefore Research Ethics Committee approval was not required.

## Results

Among the 211 consultant-led maternity units in the UK, 70% provided an incident review protocol or risk management strategy. Trusts or Health Boards that had more than one unit (n = 22) all indicated that the same guideline was in use across all units. Thus, 120 guidelines applicable to 148 maternity units were included in the appraisal. Ten of these were excerpts from protocols.

Of the English maternity units that participated in the study 64 had achieved CNST level 1, 46 were level 2, 12 were level 3 and two were ungraded. No statistically significant difference was found between the maternity units that responded and non-responders with respect to numbers of births or CNST levels (Table 1). Many of the guidelines received were maternity specific (n = 82, 68%), and the remainder applied to all incidents occurring in a Trust or Health Board irrespective of speciality. Many guidelines indicated that maternity units (n = 75, 63%) were using electronic patient safety software for reporting incidents and adverse events.

**Table 1:** **Characteristics of responding and non-responding maternity units**

	**Responded**	**Did not respond**	**p value**
No (%) of UK units	148 (70)	63 (30)	
No (%) of units in England	124 (72)	48 (28)	
No (%) of units in Northern Ireland	5 (56%)	4 (44%)	
No (%) of units in Scotland	11 (65%)	6 (35%)	
No (%) of units in Wales	8 (62%)	5 (38%)	
Median no of births per unit (UK)*	3500	3250	0.99
Range	200 to 8800	150 to 6900	
Median CNST level (Applies to units in England only)	1	1	0.46

*Number of births in UK maternity units in 2011.

**Figure 1:**
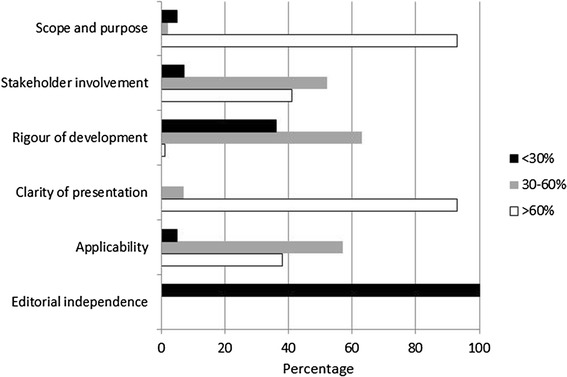
**Percentage of guidelines scoring low, medium and high in six domains of the Appraisal of Guidelines for Research and Evaluation II Instrument (AGREE II).**

The median quality scores for the six domains are shown in Table 2. Scores for the ‘scope and purpose’ domain were significantly higher than scores for the other domains (p < 0.001). The median score for this domain was 86%, and 112 of the 120 guidelines (93%) scored more than 60% (Figure 1). Only 49 guidelines scored more than 60% for the ‘stakeholder involvement’ domain which includes being authored by a range of health professionals or consulting with staff. Only one guideline scored over 60% for the ‘rigour of development’ domain, and scores were significantly less than for any other domain (p < 0.001). For the ‘clarity of presentation’ domain, 111 of 120 of guidelines (93%) scored more than 60%, and no guidelines were scored less than 30%. The median score for the ‘applicability’ domain was 56%, but only six guidelines scored less than 30%. None of the guidelines referred to the independence of the guidelines from the funding body or any competing interests of the guideline development group members.

**Table 2:** **Standardised domain scores of all guidelines (n = 120)**

**Domain**	**Median (95%** **CI)**	**Range**	**IQR**
Scope and purpose	86.1 (36.1-97.2)	0-100	77.8-91.7
Stakeholder involvement	55.6 (18.1-81.9)	0-91.7	47.2-65.3
Rigour of development	33.9 (6.8-53.6)	0-69.8	27.1-41.1
Clarity of presentation	77.8 (54.2-95.8)	47.2-100	69.4-84.7
Applicability	58.3 (30.2-84.4)	16.7-97.9	41.7-64.6
Editorial independence	0 (0-0)	0-4.2	0.0-0.0

Of the 120 guidelines that were scored, 7 (6%) had an average AGREE score of five or more and thus were classified as being of sufficiently high quality to be used in practice with no alterations. A further 56 (47%) guidelines had an average AGREE score of 4-4.9 and were recommended for use with some minor alterations, 49 (41%) were of average quality (average score of 3-3.9) and 8 (7%) were deemed to be of poor quality. The maternity units producing the recommended guidelines were not significantly different than other units who had provided guidelines in terms of numbers of births or CNST levels (p > 0.1).

Most units (n = 97, 81%) suggested the use of root cause analysis to review serious maternity incidents, with case review analysis at regular meetings for less serious incidents. The guidelines for six units did not state the approach to be used for reviewing incidents. Other approaches to review incidents that were mentioned were case reviews (10 units), significant event analysis (5 units), trend analysis (4 units), and one unit recommended choosing an approach from systems analysis with a contributory factor framework, brainstorming, the five whys, incident decision tree, fishbone diagram and gap analysis.

Most policies required one or two designated people to review reports of all incidents, such as a risk midwife or manager. Only four guidelines did not stipulate who should review incidents. Many of the other guidelines designated a team of people to review each incident including senior risk managers, lead clinicians and specialist midwives in risk. Depending on severity, incidents could be escalated to a Supervisor of Midwives, maternity risk management boards, adverse events committees, patient safety co-ordinators, relevant Executive directors, or arrangements made to have a committee with external professionals and an independent Chair. One of the highly recommended guidelines stated that incidents would be reviewed by ‘the Incidents, Complaints and Claims group which meets at a minimum six times per year,’ with other specialist staff, for example anaesthetists, being invited to attend as appropriate.

Lessons learned from incident reviews were most commonly (n = 110, 92%) disseminated at meetings, in reports, newsletters and posters, with individual feedback and support given to those involved in the incident. Other means of communication included e-mail, intranet, memos, via line managers, local and multi-disciplinary forums, in the minutes of meetings and ‘message of the week.’ Only 10 guidelines (8%) did not mention any means of communicating the findings of reviews with staff. Many guidelines (n = 83, 69%) included details of a process to audit the impact of recommendations arising from incidents. A further 28 (23%) guidelines included mention of having an auditing process or a committee that would consider governance and assurance issues, but it was not clear if the audit cycle around incidents would be completed. Only nine units that responded made no mention of audits.

## Discussion

Just over half of the local NHS maternity incident review guidelines were of a good or high standard. However, many guidelines did not score highly for the ‘Stakeholder involvement’ and ‘Rigour of development’ domains of the AGREE criteria. Root cause analysis methodology was advised to review cases in more than four fifths of units, although several units simply recommended the use of case reviews. Risk midwives or managers were usually designated to review reports of all incidents, with the option to escalate to other professionals. Almost all guidelines suggested various means to disseminate lessons learned to staff, but over a quarter of guidelines did not stipulate the auditing of changes made in clinical practice arising from maternity incidents.

This evaluation was based on 120 guidelines applicable to 148 (70%) of the 211 maternity units in the UK. The response rate was high, and there were no systematic differences between the units that responded and those that did not in terms of numbers of births or CNST level. These findings contrast with those from an appraisal of guidelines for the transfer of women in labour to obstetric units, which found local guidance to be of poor quality and that better guidelines were produced by units with higher numbers of births [18].

We are unable to make any judgements about the use or quality of guidelines in maternity units that did not respond. For 10 units only excerpts of guidelines were received instead of whole documents, which may have reduced their domain scores. Two appraisers reviewed the guidelines and gave scores that were largely in agreement for each AGREE II item. Having additional appraisers may have increased the reliability of the instrument, but could be unnecessary given the similarity between the independent scores. We chose to discuss the questions in the AGREE criteria and inconsistencies in scoring before agreeing final scores. It is not standard practice to discuss the questions or review scores, but these measures were useful given that neither appraiser had used the AGREE instrument before, some questions could have been interpreted as being repetitive, and there were some errors and discrepancies in interpretation. Following this discussion, the application of the AGREE II instrument was straightforward.

The AGREE II instrument was supplemented by analysing methods used for reviewing incidents, the people to be involved in a review and the methods for disseminating outcomes, because there is no proven link between the quality of a guideline as determined by the AGREE II appraisal, and the quality of the content of a guideline. An overall mean average score was calculated for each guideline and then guidelines were categorised as poor, average, good or high quality to provide an objective, quantitative measure of the quality of a guideline. Current guidance suggests a more subjective approach that we found did not capture variability in quality between guidelines [19].

The pattern of higher scores for the ‘scope and purpose’ and ‘clarity of presentation’ domains, and lower scores for the ‘stakeholder involvement’, ‘rigour of development’ and ‘applicability’ domains is similar to that found in two other appraisals of local guidelines [18,20]. One of these studies did not use the ‘editorial independence’ domain and in the other only minimum scores were recorded. It is of concern that not one single guideline recorded or addressed the competing interests of guideline development members, thus leading to minimum scores being recorded for ‘editorial independence’.

Overall, the guidelines scored particularly well on the ‘scope and purpose’ and ‘clarity and presentation’ domains, often citing guidance to ‘being open’ with patients and families and supporting staff involved in incidents from the Department of Health [1], Royal College of Obstetricians and Gynaecologists [6] and the NHSLA [2].

The ‘rigour of development’ domain was poorly scored. Most guidelines contained no information on the methods used for development and did not refer to specific evidence underpinning recommendations. However, many guidelines referred to national reports and policies indicating that guidance is likely to have been adapted for local use [1,2,6,13]. Failure to include a score for the use of national guidance positively in the ‘rigour of development’ domain may be regarded as a potential limitation of the AGREE II instrument. It could be generally questioned whether the AGREE II instrument is suitable for appraising adaptations of (national) guidelines.

The ‘stakeholder involvement’ domain was not scored highly because many guidelines had only been drafted by one or two individuals. Better scores were achieved for guidelines that were reviewed by clinical governance or risk management committees and other stakeholders. Some units circulated the guidance to staff to obtain their views, but it was rare for service users to be invited to comment. No guidelines recommended that maternity service users should be included on incident review panels. Qualitative studies of the experiences of women and their partners indicate that negative experiences were characterised by powerlessness and exclusion [21,22]. Careful consideration could therefore be given to the involvement of members of the public in the formulation of guidance and reviews of maternity incidents.

The ‘applicability’ domain was also not scored highly. Many guidelines included flowcharts on the protocol for reviewing incidents, and mentioned appropriate education and training for staff. Few described facilitators or barriers to application or considered the resources (people, finance and time) needed for conducting incident reviews. Only one of the recommended guidelines explicitly stated that managers taking part in reviews may need to delegate other pressing duties.

Root cause analysis was used by most units to review serious incident cases, as originally recommended by the National Patient Safety Agency [17]. The quality of serious maternity incident reviews has been questioned in the past [4], and it is of concern that a small number of units still did not stipulate any robust methodology for reviewing incidents, despite national guidance being in existence since 2008. Several units provided hospital-wide policies for reviewing incidents. Maternity-specific policies were assessed to be of higher quality because they stipulated that appropriate professionals, such as midwives, obstetric anaesthetists and obstetricians, be involved in the review process. Case review was often cited as the method to be used for less serious cases, although the specifics of this approach remain ambiguous. Many guidelines included monitoring the impact of changes in clinical practice arising from incidents, but audits of care should be standard practice. The assessment of quality should not merely focus on changes in structures and processes, but should include monitoring outcomes [23].

## Conclusion

Overall, local guidance for the review of maternity incidents was mostly of good or high quality. A small number of units had no guidance. Two main areas were identified by AGREE criteria which could be improved. Stakeholder participation could be widened, such that professionals from different backgrounds as well as service users should contribute to the development of maternity guidelines and reviews of incidents. Editorial independence was frequently unclear; competing interests of guideline developers should be clearly stated. Additionally, it was unclear in over a quarter of guidelines whether changes in practice in response to review recommendations were audited or monitored; such auditing should be mandatory. Further research is required to examine the translation of guidance into practice by evaluating the quality of local reviews of maternity incidents.
